# Factors Associated With Psychosocial Functioning and Outcome of Individuals With Recent-Onset Schizophrenia and at Ultra-High Risk for Psychosis

**DOI:** 10.3389/fpsyt.2019.00459

**Published:** 2019-06-26

**Authors:** Hyun Kyu Kim, Hye Yoon Park, Eunchong Seo, Minji Bang, Yun Young Song, Su Young Lee, Kyung Ran Kim, Jin Young Park, Jee In Kang, Eun Lee, Suk Kyoon An

**Affiliations:** ^1^Department of Psychiatry, Yonsei University College of Medicine, Severance Hospital, Seoul, South Korea; ^2^Section of Self, Affect and Neuroscience, Institute of Behavioral Science in Medicine, Yonsei University College of Medicine, Seoul, South Korea; ^3^Department of Psychiatry, CHA Bundang Medical Center, CHA University, Seongnam, South Korea; ^4^Department of Psychiatry, Myongji Hospital, Hanyang University College of Medicine, Goyang, South Korea; ^5^Department of Psychiatry, Yonsei University College of Medicine, Gangnam Severance Hospital, Seoul, South Korea; ^6^Graduate Program in Cognitive Science, Yonsei University, Seoul, South Korea

**Keywords:** conversion, readmission, psychosocial function, schizophrenia, ultra-high risk for psychosis

## Abstract

**Background:** Patients with schizophrenia have impairments in social functioning and are readmitted to healthcare institutions frequently. Individuals at ultra-high risk (UHR) for psychosis already present poor social functioning; among those individuals, the conversion rate from the putative prodromal phase to overt psychosis is 20%–30% within 1–2 years. Here, we analyzed the factor structure of self-related variables and neuro- and socio-cognitive function, and investigated whether these factors were associated with psychosocial function and prognostic outcome in individuals with recent-onset schizophrenia (ROSPR) or at UHR for psychosis.

**Methods:** We evaluated 60 individuals at UHR for psychosis, 47 individuals with ROSPR, and 71 healthy controls using a comprehensive neurocognitive test battery and self-reported attribution scales, self-esteem, resilience, aberrant subjective experiences of schizotypy (physical anhedonia, social anhedonia, magical ideation, and perceptual aberration), and basic symptoms. We assessed psychosocial function with the Quality of Life Scale (QLS).

**Results:** Factor analysis of all subjects revealed a four-factor structure comprising social-cognitive bias, reflective self, neurocognition, and pre-reflective self factors. Multiple regression analysis at baseline revealed that the factor structure predicted QLS. In the UHR group, social-cognitive bias, reflective self, neurocognition, and negative symptoms were significant determinants, explaining 38.0% of total QLS score variance. In the ROSPR group, reflective self and negative symptoms were significant determinants, explaining 54.4% of total QLS score variance. During follow-up, 13 individuals at UHR for psychosis developed psychosis (cumulative prevalence: 31.2% ± 7.6% at 6 years), with neurocognition score at baseline remaining a significant predictor of conversion [χ^2^(1) = 4.009, *p* = 0.045; hazard ratio 0.56, 95% confidence interval 0.31–0.99, *p* = 0.048]. Five patients with schizophrenia were (re)admitted during follow-up (cumulative prevalence: 16.1% ± 7.1% at 6 years); no factor was found to predict (re)admission.

**Conclusion:** Factor analysis revealed an intrinsic four-factor structure of social-cognitive bias, reflective self, neurocognition, and pre-reflective self. The four factors were associated with social functioning at baseline and prodrome-to-psychosis conversion during follow-up, indicating the clinical significance of the four-factor structure. These findings provide a framework for understanding schizophrenia.

## Introduction

Patients with schizophrenia have impairments in social functioning and are readmitted to healthcare institutions frequently. Declining social functioning, a hallmark of schizophrenia, occurs throughout the course of the disorder and may begin even prior to overt psychotic symptoms (1). Frequent readmission to healthcare institutions affects not only social functioning but also the quality of life of patients with schizophrenia. Understanding the factors associated with impaired social functioning and readmission to a healthcare institution may be crucial to help patients with schizophrenia achieve better quality of life.

Vulnerability factors of patients with schizophrenia include impaired neurocognition and social cognition. Neurocognitive impairment is related to a decline in social functioning, and verbal memory (2), spatial organization (3), visual memory, and intelligence quotient (4) have been suggested to correlate with social and vocational outcome in patients with schizophrenia (5). Poor performance on the Wisconsin card sorting test has been suggested as a predictor of rehospitalization in patients with schizophrenia, even after controlling for adherence to medication (5, 6). Social cognition impairment has also been suggested to be associated with social functioning. Impaired facial affect recognition is correlated with social functioning in patients with first- or multi-episode schizophrenia (7). There is also a correlation between impaired social perception and role functioning in patients with schizophrenia (8), and attribution bias has been found to have an effect on social functioning impairment in patients with bipolar disorder and schizophrenia (9). A recent meta-analysis showed that while overall impairments in social cognition are more strongly correlated with community functioning than impaired neurocognition is, cognitive functioning only explains 25% of outcome variance in patients with schizophrenia (10). Besides the abovementioned objective cognitive deficits, patients with schizophrenia experience subjective symptoms, including basic symptoms (11) and schizotypy (12), both of which are suggested to result from deficits in information processing (13). Previous longitudinal studies suggested that basic symptoms have a negative relationship with social functioning and quality of life in patients with schizophrenia (14) and that schizotypy predicts social functional impairment (15). One review article suggested that basic symptoms are associated not only with disease itself but also with relapse of schizophrenic episodes; since basic symptoms occur prior to relapse, they are considered an early sign of relapse (16). Schizotypy is associated with dopamine changes (17). It occurs in patients genetically prone to psychotic episodes (18). Therefore, schizotypy may affect schizophrenia relapses (19) and be associated with rehospitalization.

Regarding the protective factors, resilience has been associated with social functioning in patients with schizophrenia (20, 21). Self-esteem has also been reported to correlate with social functioning in psychiatric outpatients, including those with schizophrenia (22). Patients with schizophrenia who are less resilient have more frequent and more severe episodes, including rehospitalization (23). Low self-esteem of patients with schizophrenia is associated with stigma (24) and higher rehospitalization rates (25).

Ultra-high risk (UHR) for psychosis is a putative prodromal phase when poor social functioning is already present. The conversion rate to overt psychosis is around 30% during the follow-up period (26). It is important to find the factors associated with social functioning and conversion to overt psychosis in individuals at UHR for psychosis. Declining neurocognitive function has been reported to be associated with social functioning impairment (27, 28) and psychotic conversion (29–33) in individuals at UHR for psychosis. Neurocognitive functions, such as verbal learning, memory, processing speed, attention, and verbal fluency, predict social functioning outcome in UHR for psychosis (34). Spatial memory is one of the factors that significantly predict conversion (29, 30). Working memory and verbal ability deficits are possible predictors of psychotic conversion in initial prodromal states (32). In line with these observations, working memory, visual memory, and executive function have been included in the psychotic conversion model of individuals at UHR for psychosis (31). Social cognition, including the theory of mind, is impaired in individuals at UHR for psychosis (35, 36); it plays an important role in social functioning impairment (37) and predicts psychotic conversion (31). Resilience is also an important influencing factor of social functioning in individuals at UHR for psychosis. Individuals with higher resilience present better psychosocial functioning (38). Conversely, the conversion rate is higher in low-resilience patients (38). Basic symptoms and schizotypy of physical anhedonia are other predicting factors of conversion in individuals at UHR for psychosis (39).

As mentioned above, psychosocial functioning and readmission of patients with schizophrenia or psychotic conversion in individuals at UHR for psychosis are associated with loss of the ability to form complex and integrated ideas and experiences of the self and others. This ability may include verbal memory, spatial memory, facial affect recognition, theory of mind, attribution style, resilience, basic symptoms, and schizotypy as mentioned previously. In our previous study, we concluded these factors can be categorized as cognitive and self-related factors (40). Cognitive factors include neurocognitive factors (spatial memory, verbal memory, intelligence quotient, etc). and social cognitive functions (facial affect recognition, theory of mind, attribution style, etc). Self-related factors include resilience, self-esteem, basic symptoms, and schizotypy; they can be subcategorized as two levels of self: pre-reflective and reflective levels (40). These two levels of concept of self were firstly introduced as phenomenological theory for understanding self-experience. Furthermore, the underlying neural underpinnings of these two levels of self were found to be dissociated from each other. For example, Esslen et al. (41) revealed biological evidence that ventral parts of medial prefrontal cortex were related to pre-reflective self and dorsal parts of medial prefrontal cortex were related to reflective self. The pre-reflective self is a first-person perspective and minimal level of self; it is also called basic self, minimal self, or ipseity (41). This aspect is a result of direct and non-reflective experiencing of self. Basic symptoms and schizotypy, such as perceptual aberrations and magical ideation, are measures of the pre-reflective level of self. In contrast, the reflective level of the self is a result of self-introspection and explicit awareness of the self; it is also regarded as the narrative self (42). Since the reflective self includes all aspects of an individual’s personality (42), self-esteem and resilience can reflect this level of self. Previous studies provided disseminated information of relationships among these aspects with social functioning/readmission of patients with schizophrenia and social functioning/conversion of individuals at UHR for psychosis, but there was no study that integrates and categorizes the cognitive and self-related factors associated with functioning and prognostic outcome. Thus, we wanted to find and construct a proper factor structure of cognitive and self-related factors that is associated with social functioning/readmission of patients with schizophrenia and social functioning/conversion of individuals at UHR for psychosis. This factor structure provides an integrated perspective to understand social functioning/readmission of patients with schizophrenia and social functioning/conversion of individuals at UHR for psychosis.

The aims of this study were to analyze the factor structure of self-related psychosocial variables and cognitive function and investigate whether these factors were associated with social function and prognostic outcome in individuals with recent-onset schizophrenia (ROSPR) or at UHR for psychosis. We hypothesized that i) cognitive function and self-related variables can be categorized into representable factors in all subjects, and these factors contain the characteristics of neurocognitive function, social-cognitive bias, reflective self, and pre-reflective self; ii) these factors are significantly different among the UHR, ROSPR, and control groups; and iii) these factors are associated with psychosocial function at baseline in the UHR and ROSPR groups, with the psychotic conversion rate, and with the (re)admission rate during follow-up in the UHR and ROSPR group, respectively.

## Materials and Methods

### Subjects

We included 60 individuals at UHR for psychosis, 47 individuals with ROSPR, and 71 healthy controls (HCs) in this study. The HC group was recruited through online advertising between July 2007 and September 2016; subjects with any past or current psychiatric or neurological illness were excluded. The UHR and ROSPR groups comprised help-seeking individuals recruited at the early psychosis clinic (Clinic FORYOU) at Severance Hospital of Yonsei University Health System in the Seoul metropolitan area during the same period. In all subjects, axis I psychiatric disorders were assessed by a trained psychiatrist (K.K.R.) using the Structured Clinical Interview for DSM-IV (SCID-IV) (43, 44). Individuals at UHR for psychosis were diagnosed according to the criteria of the Structured Interview for Prodromal Syndromes (SIPS) (45). To be diagnosed with UHR for psychosis, individuals had to satisfy one or more of the following prodromal syndromes outlined in the SIPS: 1) brief intermittent psychotic syndrome (BIPS), which has emerging psychotic symptoms with spontaneous remission in less than 1 week; 2) attenuated positive prodromal syndrome (APS), which has attenuated subthreshold positive psychotic symptoms; and/or 3) genetic risk and deterioration syndrome (GRDS), which is a combination of genetic risk for schizophrenia and recent functional decline. After inclusion in the study, individuals in the UHR group were re-assessed every month for 24 months and at regular outpatient follow-up intervals after 24 months by the psychiatrist-in-chief (A.S.K.) to determine whether the conversion to overt psychosis had occurred. Conversion to overt psychosis was defined when the patient met the DSM-IV criteria for psychotic disorders, including schizophrenia, schizoaffective disorder, psychotic disorder not otherwise specified, and mood disorders with psychotic features. ROSPR was diagnosed according to the criteria of the DSM-IV using the SCID-IV. At baseline, patients with ROSPR were limited to those who had experienced their first (*n* = 42) or second (*n* = 5) psychotic episode within less than 36 months from the first frank psychotic episode. Of the 47 patients with ROSPR, 17 were inpatients, 9 were outpatients with a history of one psychiatric hospitalization, and 21 were outpatients without a history of hospitalization. After inclusion in the study, individuals in the ROSPR group were re-assessed every month for 24 months and at regular outpatient follow-up intervals after 24 months by the psychiatrist-in-chief (A.S.K.) to determine whether psychiatric (re)admission had occurred due to relapse of psychotic episodes.

This study was carried out in accordance with the Declaration of Helsinki. The Institutional Review boards at Severance Hospital reviewed and approved this study. All subjects, or the parents of subjects who were under 18 years old, gave written informed consent to participate in the study.

### Measures

#### Cognitive Variables

We assessed the neurocognitive function of the subjects using a comprehensive neurocognitive test battery, as described in our previous study (28). The battery comprises the Rey Complex Figure Test (46), California Verbal Learning Test (47), 3–7 Continuous Performance Test (48), Controlled Oral Word Association Test (49), Figure Fluency Test (50), Trail Making Test Part A and B (51), Verbal and Spatial 2-back Test (52), Stroop Test (53), and Wisconsin Card Sorting Test (54). The *z* scores were converted from each neurocognitive test score based on the performance of the HC group (*n* = 94) (29). These scores were categorized into five dimensions representing the factor structure determined previously (28): verbal memory, spatial memory, psychomotor speed, attention/working memory, and executive function. Summary scores for each dimension were calculated as the mean of the test scores in that same category. The internal consistencies of these five dimensions of neurocognitive function were good (for verbal memory, Cronbach’s alpha = 0.891; for spatial memory, Cronbach’s alpha = 0.975; for attention/working memory, Cronbach’s alpha = 0.807) in the entire groups of subjects, except psychomotor speed (Cronbach’s alpha = 0.635) and executive function (Cronbach’s alpha = 0.594).

We assessed social-cognitive bias using the Ambiguous Intentions Hostility Questionnaire (AIHQ) (55, 56). The AIHQ is a self-report checklist of 15 hypothetical negative situations. The situations vary in intentionality: five are accidental, five are ambiguous, and five are intentional situations. The AIHQ yields scores of hostility, aggression bias, and composite blame bias. The hostility and aggression biases are rated by the rater based on the participant’s written response according to the sample scores for each item, provided in the AIHQ scoring form. The composite blame score is the mean of intent, anger, and blame scores. In this study, we only used the hostility and composite blame scores for each hypothetical situation. The validity of each score was good in blame intentional (Cronbach’s alpha = 0.840) and blame accidental (Cronbach’s alpha = 0.820). Acceptable internal consistencies were found in blame ambiguous (Cronbach’s alpha = 0.770), hostility ambiguous (Cronbach’s alpha = 0.745), and hostility accidental (Cronbach’s alpha = 0.777), except for hostility intentional (Cronbach’s alpha = 0.641).

#### Self-Related Variables

Self-related measures comprised self-esteem [Rosenberg’s Self-esteem Scale (57)], resilience [Connor–Davidson Resilience Scale (58)], features of schizotypy [Chapman’s true-false self-report questionnaires for social anhedonia (59), physical anhedonia (60), perceptual aberration (61), and magical ideation (62)], and basic symptoms [Frankfurt Complaint Questionnaire (63)]. The internal consistencies of these self-related variables were good (for self-esteem, Cronbach’s alpha = 0.898; for resilience, Cronbach’s alpha = 0.937; for social anhedonia, Cronbach’s alpha = 0.926; for physical anhedonia, Cronbach’s alpha = 0.926; for perceptual aberration, Cronbach’s alpha = 0.896; for magical ideation, Cronbach’s alpha = 0.835; for basic symptom, Cronbach’s alpha = 0.976).

#### Psychopathology

We assessed symptom severity using the Scale for the Assessment of Positive Symptoms (SAPS) (64) and the Scale for the Assessment of Negative Symptoms (SANS) (65).

#### Psychosocial Function

We assessed psychosocial function using the Heinrichs–Carpenter Quality of Life Scale (QLS) (66). The QLS is a rater-administered scale with 21 items, each scoring 0–6 points. The result of the QLS reveals the total score and the scores of its four subscales: interpersonal relations, instrumental role, intrapsychic foundation, and common objects and activities (38). The Korean version of the scale has been widely used in studies of social functioning in schizophrenia (67).

### Statistical Analysis

We used univariate analysis of variance (ANOVA) and χ^2^ tests to compare the differences in demographic and clinical characteristics among the three groups. In addition, we performed exploratory factor analysis to categorize the measures to establish the factor structure. We applied exploratory factor analysis, not confirmative analysis, due to the absence of factor structure information from previous studies. A scree plot and factors with eigenvalues >1 were used to determine the number of factors. After varimax rotation, items with factor loading ≥0.4 were considered to be significant. Factor scores were derived by weighted sum of each variable score with factor loadings for use in multiple regression analysis. Items with two significant factor loadings were assigned to the factor with higher loading. We compared the factor scores among the three groups using ANOVA and analysis of covariance (ANCOVA) with age and education years as covariates, followed by *post hoc* analysis with Bonferroni correction. Statistical significance was set at *p* < 0.05.

We used Kaplan–Meier survival analysis to determine the cumulative rate of conversion from UHR for psychosis to overt psychosis and the (re)admission rate of patients with ROSPR. We used Cox regression analysis to estimate possible predictors for conversion in the UHR group and (re)admission in the ROSPR group.

## Results

### Subject Characteristics

The demographic and clinical characteristics of each group are presented in **Table 1**. The average duration of illness in the ROSPR group was 11.4 months.

**Table 1:** Baseline demographic and clinical characteristics of the study groups.

	HC (*n* = 71)	UHR (*n* = 60)	ROSPR (*n* = 47)	Statistical analysis			
				Value	*p*	Post hoc	p*
**Age (years)**							
** Mean (SD)**	22.0 (3.4)	20.3 (3.5)	23.0 (4.1)	*F* = 6.6	0.002	H vs. U	0.038
						H vs. S	0.59
						U vs. S	0.002
** Range** **^†^**	15–28	16–28	15–35				
** Gender (male/female)**	34/37	34/26	21/26	χ^2^ = 1.725	0.422		
**Education**							
** Years (SD)**	13.9 (1.8)	13.0 (1.9)	13.6 (2.0)	*F* = 3.85	0.023	H vs. U	0.02
						H vs. S	1.0
						U vs. S	0.26
**SAPS (summary score)**	0.014 (0.12)	3.62 (2.39)	6.17 (2.90)	*F* = 135.93	<0.001	H vs. U	<0.001
						H vs. S	<0.001
						U vs. S	<0.001
**SANS (summary score)**	0.54 (1.26)	8.32 (4.11)	8.89 (5.27)	*F* = 99.96	<0.001	H vs. U	<0.001
						H vs. S	<0.001
						U vs. S	<0.001
**Number of episodes**							
** 1st episode**			42				
** 2nd episode**			5				
**Types of UHR**							
** APSS only**		43					
** BIPS only**		1					
** GRDS only**		0					
** APSS + BIPS**		5					
** APSS + GRDS**		11					
**Duration of illness (months)**			11.4 (11.4)				
**Antipsychotic medication status**							
** Medicated/unmedicated**		18/42	45/2	χ^2^ = 47.05	<0.001		
**Chlorpromazine equivalent (mg/day)** **^‡^**		114.0 (95.2)	433.42 (323.09)		<0.001		

HC, healthy control; UHR, ultra-high risk for psychosis; ROSPR, recent-onset schizophrenia; SAPS, Scale for the Assessment of Positive Symptoms; SANS, Scale for the Assessment of Negative Symptoms; APSS, attenuated positive symptoms syndrome; BIPS, brief intermittent psychotic syndrome; GRDS, genetic risk and deterioration syndrome.

*Corrected p values are derived from post hoc comparisons with Bonferroni correction.

^†^Ranges presented in this table were 5–95 percentile age of each group.

^‡^All medicated ROSCR and UHR participants were taking atypical antipsychotic medications (68).

### Factor Analysis of Cognitive and Self-Related Variables

**Table 2** presents the results of exploratory factor analysis with varimax rotation. Eighteen variables were reduced to four factors with eigenvalues of 5.89, 2.55, 1.70, and 1.42, respectively. A Kaiser–Meyer–Olkin (KMO) value of 0.79 confirmed sampling adequacy, and Bartlett’s test of sphericity was statistically significant (*p* < 0.001). High loadings on factor 1 were mainly from AIHQ scores. High loadings on factor 2 were mainly from high physical/social anhedonia and less self-esteem and resilience. High loadings on factor 3 were from all the neurocognitive tests. High loadings on factor 4 were from high magical ideation, perceptual aberration, and basic symptom scores. Considering the high loadings on each of the factors 1–4, they were named social-cognitive bias, reflective self, neurocognition, and pre-reflective self, respectively.

**Table 2:** Loadings on factors derived by exploratory factor analysis with varimax rotation.

	Factor 1: Social-cognitive bias	Factor 2: Reflective self	Factor 3: Neurocognition	Factor 4: Pre-reflective self
**Blame ambiguous**	0.766*	0.284	0.027	0.231
**Hostility accidental**	0.765*	0.1	−0.199	−0.009
**Blame accidental**	0.763*	0.027	−0.133	0.237
**Blame intentional**	0.709*	0.1	0.178	0.020
**Hostility intentional**	0.689*	0.237	0.062	−0.014
**Hostility ambiguous**	0.588*	0.433*	−0.033	0.171
**Self-esteem**	−0.232	−0.819*	0.052	−0.192
**Resilience**	−0.250	−0.809*	0.136	−0.100
**Physical anhedonia**	0.097	0.790*	−0.060	0.145
**Social anhedonia**	0.272	0.772*	−0.084	0.260
**Magical ideation**	0.151	0.140	−0.080	0.871*
**Perceptual aberration**	0.078	0.170	−0.109	0.837*
**Basic symptoms**	0.164	0.460*	−0.119	0.759*
**Verbal memory**	−0.074	−0.024	0.774*	−0.111
**Attention/working memory**	0.017	−0.179	0.738*	−0.131
**Psychomotor speed**	0.035	−0.361	0.674*	0.035
**Executive function**	−0.062	0.063	0.629*	−0.145
**Spatial memory**	0.037	0.015	0.603*	0.052

All loadings are represented such that positive scores indicate higher scores on the item. *Loadings > 0.40.

### Comparison of Baseline Social Cognitive Bias, Reflective Self, Neurocognition, and Pre-Reflective Self Factors among the UHR, ROSPR, and HC Groups

There were significant differences among the three groups in reflective self, neurocognition, and pre-reflective self factors, but not in the social cognitive bias factor (**Table 3**). Post hoc analysis with Bonferroni correction revealed that the UHR group had the highest reflective self factor score, followed by the ROSPR and HC groups. The neurocognition factor score of the ROSPR group was significantly poorer than that of the UHR (*p* < 0.001) and HC (*p* < 0.001) groups, and there was no significant difference between the UHR and HC groups (*p* = 0.096). The pre-reflective self factor score of the HC group was significantly lower than that of the UHR (*p* = 0.004) and ROSPR (*p* = 0.03) groups, and there was no significant difference between the UHR and ROSPR groups (*p* > 0.999). Results from ANCOVA with age and education year as covariates revealed a significant interaction between education year and the neurocognition factor (*p* = 0.014); no other significant interactions were found.

**Table 3:** Factor scores of each group.

	HC (*n* = 71)	UHR (*n* = 60)	ROSPR (*n* = 47)	Statistical analysis			
				Value	*p*	Post hoc	Corrected *p* values*
**Factor 1:** **Social-cognitive bias**	−0.096 (0.59)	0.16 (1.17)	−0.063 (1.24)	*F* = 1.04	0.378	H vs. U	0.588
						H vs. S	1.0
						U vs. S	1
**Factor 2:** **Reflective self**	−0.68 (0.66)	0.71 (0.95)	0.12 (0.80)	*F* = 33.9	<0.001	H vs. U	<0.001
						H vs. S	<0.001
						U vs. S	0.004
**Factor 3:** **Neurocognition**	0.47 (0.50)	0.11 (0.84)	−0.85 (1.20)	*F* = 23.9	<0.001	H vs. U	0.096
						H vs. S	<0.001
						U vs. S	<0.001
**Factor 4:** **Pre-reflective self**	−0.32 (0.71)	0.28 (1.10)	0.13 (1.13)	*F* = 5.15	0.002	H vs. U	0.004
						H vs. S	0.028
						U vs. S	1.0

HC, healthy control; UHR, ultra-high risk for psychosis; ROSPR, recent-onset schizophrenia.

*Corrected p values are derived from post hoc comparisons with Bonferroni correction.

### Associations Between Baseline Factor Structure and Qls in the Uhr Group

In the UHR group, SANS score and social-cognitive bias, reflective self, and neurocognition factors were significant determinants explaining 38.0% of the total QLS score variance (**Table 4**). SANS score and reflective self and neurocognition factors accounted for 27.2% of variance in the regression model of interpersonal relations, with statistical significance. Social-cognitive bias and neurocognition factors were significant determinants in the regression model of instrumental role, with an explanatory power of 22.6%. For the intrapsychic foundation, SANS score and social-cognitive bias, reflective self, and neurocognition factors were significant determinants accounting for 46.0% of variance. Common objects and activities of QLS had no significant predictors in the regression analysis. When initial antipsychotics dose was treated as a covariate, the results did not change.

**Table 4:** Multiple regression analysis to predict QLS from factor structure.

	Dependent variable	Independent variables	B	SE	β	*t*	*P*	Model’s properties
UHR (*n* = 60)	Total score of QLS	(Constant)	70.100	5.355		13.091	<0.001	*R* ^2^ = 0.443, adj. *R* ^2^ = 0.380, *F* = 7.027, *p* < 0.001
		SANS	−1.585	0.560	−0.323	−2.830	0.007	
		Social-cognitive bias	−4.079	1.839	−0.236	−2.287	0.026	
		Reflective self	−5.852	2.559	−0.275	−2.287	0.026	
		Neurocognition	9.295	2.857	0.388	3.253	0.002	
	Interpersonal relations of QLS	(Constant)	23.973	2.657		9.024	<0.001	*R*^2^ = 0.346, adj. *R*^2^ = 0.272, *F* = 4.677, *p* = 0.001
		SANS	−0.728	0.278	−0.324	−2.619	0.011	
		Reflective self	−2.845	1.270	−0.292	−2.241	0.029	
		Neurocognition	3.851	1.417	0.351	2.717	0.009	
	Instrumental role of QLS	(Constant)	2.410	0.436		5.522	<0.001	*R* ^2^ = 0.303, adj. *R* ^2^ = 0.226, *F* = 3.867, *p* = 0.003
		Social-cognitive bias	−0.415	0.150	−0.329	−2.770	0.008	
		Neurocognition	0.706	0.233	0.405	3.032	0.004	
	Intrapsychic foundation of QLS	(Constant)	26.074	1.706		15.285	<0.001	*R* ^2^ = 0.515, adj. *R* ^2^ = 0.460, *F* = 9.389, *p* < 0.001
		SANS	−0.471	0.178	−0.281	−2.641	0.011	
		Social-cognitive bias	−1.651	0.586	−0.280	−2.818	0.007	
		Reflective self	−2.897	0.815	−0.398	−3.553	0.001	
		Neurocognition	3.447	0.910	0.422	3.787	<0.001	
	Common objects and activities of QLS	(Constant)	8.233	0.727		11.318	<0.001	*R*^2^ = 0.146, adj. *R*^2^ = 0.050, *F* = 1.514, *p* = 0.192
ROSPR (*n* = 47)	Total score of QLS	(Constant)	83.543	6.142		13.602	<0.001	*R*^2^ = 0.603, adj. *R*^2^ = 0.544, *F* = 10.135, *p* < 0.001
		SANS	−3.008	0.464	−0.718	−6.488	<0.001	
		Reflective self	−6.981	2.949	−0.254	−2.367	0.023	
	Interpersonal relations of QLS	(Constant)	31.487	3.033		10.383	<0.001	*R*^2^ = 0.533, adj. *R*^2^ = 0.463, *F* = 7.605, *p* < 0.001
		SANS	−1.252	0.229	−0.656	−5.467	<0.001	
		Reflective self	−3.355	1.456	−0.268	−2.304	0.027	
	Instrumental role of QLS	(Constant)	3.065	0.523		5.856	<0.001	*R*^2^ = 0.203, adj. *R*^2^ = 0.083, *F* = 1.696, *p* = 0.147
	Intrapsychic foundation of QLS	(Constant)	32.506	2.571		12.643	<0.001	*R*^2^ = 0.564, adj. *R*^2^ = 0.499, *F* = 8.624, *p* < 0.001
		SANS	−1.067	0.194	−0.637	−5.495	<0.001	
		Reflective self	−3.370	1.235	−0.307	−2.730	0.009	
	Common objects and activities of QLS	(Constant)	7.920	0.878		9.023	<0.001	*R* ^2^ = 0.180, adj. *R* ^2^ = 0.057, *F* = 1.459, *p* = 0.217

HC, healthy control; UHR, ultra-high risk for psychosis; ROSPR, recent-onset schizophrenia; SAPS, Scale for the Assessment of Positive Symptoms; SANS, Scale for the Assessment of Negative Symptoms.

### Associations Between Baseline Factor Structure and QLS in the ROSPR Group

In the ROSPR group, SANS score and the reflective self factor were significant determinants explaining 54.4% of the total QLS score, 46.3% of the interpersonal relations score, and 49.9% of the intrapsychic foundations score (**Table 4**). Regression analysis revealed no significant predictors of common objects, activities and instrumental role in the ROSPR group. Treating initial antipsychotics dose as a covariate did not change the results.

### Conversion From UHR for Psychosis to Overt Psychosis During Follow-Up and Its Predictive Factors

**Figure 1:**
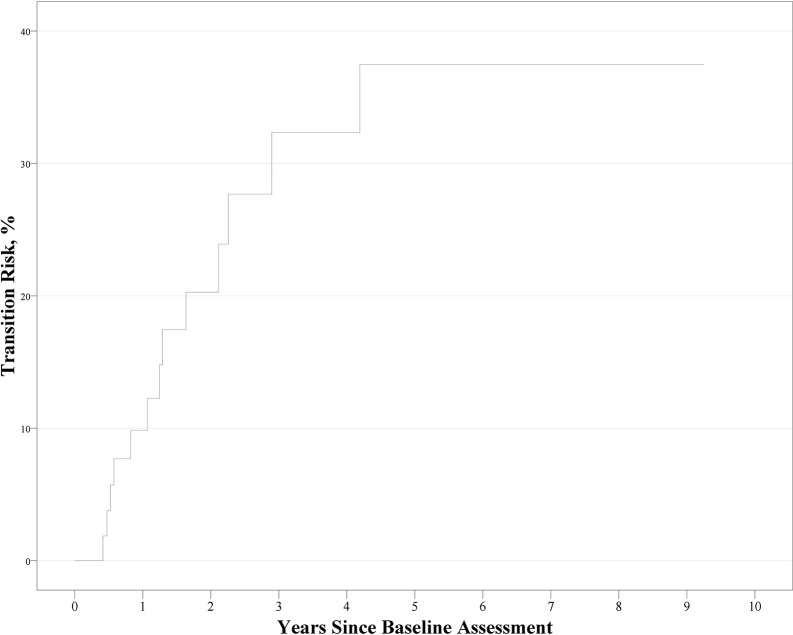
Kaplan–Meier curve for the conversion to psychosis in ultra-high risk participants (*n* = 60).

During follow-up, 13 cases of UHR for psychosis converted to overt psychosis. The cumulative prevalence rate and standard error from Kaplan–Meier estimates was 9.4% ± 4% at 1 year, 18.3% ± 5.6% at 2 years, and 31.2% ± 7.6% at 6 years. The Kaplan–Meier curve is shown in **Figure 1**. Cox regression analysis to evaluate the hazard ratio of each of the four factors revealed that only neurocognition factor score remained significant as a predictor for conversion [χ^2^(1) = 4.009, *p* = 0.045; hazard ratio, 0.56; 95% confidence interval, 0.31–0.99; *p* = 0.048].

### (Re)admission of Patients With ROSPR During Follow-Up and Its Predictive Factors

**Figure 2:**
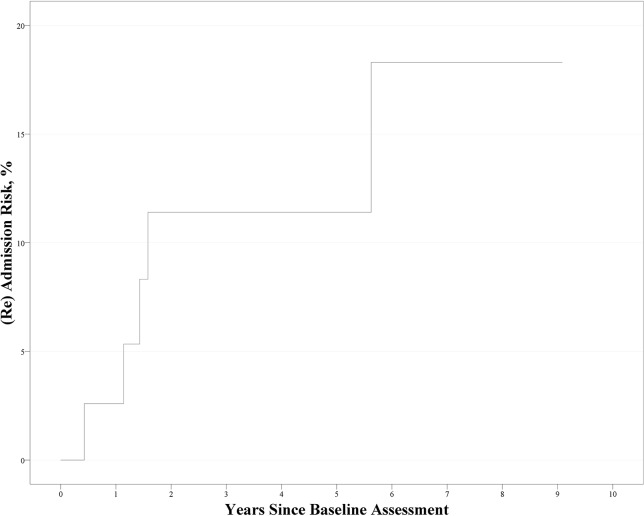
Kaplan–Meier curve for the (re)admission of recent-onset schizophrenia patients (*n* = 47).

During follow-up, five patients with ROSPR were (re)admitted to the hospital. The Kaplan–Meier curve is shown in **Figure 2**. The cumulative prevalence rate and standard error from Kaplan–Meier estimates were 2.5% ± 2.5% at 1 year, 10.5% ± 5.0% at 2 years, and 16.1% ± 7.1% at 6 years. Cox regression analysis revealed no significant factor affecting the (re)admission rate [χ^2^(1) = 2.630, *p* = 0.105].

## Discussion

In the present study, we identified a four-factor structure of social cognitive bias, reflective self, neurocognition, and pre-reflective self in UHR, ROSPR, and HC individuals. There were overall group differences in these four factors between the UHR, ROSPR, and HC groups. Importantly, these factors were found to be associated with baseline psychosocial function in the UHR and ROSPR groups as well as with conversion rate in the UHR group during follow-up.

The four-factor structure contained several self-related and cognitive variables. Scores from AIHQ subsets were categorized as one factor named social cognitive bias, which is one of the components of social cognition and a measure of social cognition in previous studies (69, 70). Among the self-related factors, two distinct factors were found: self-esteem, resilience, physical anhedonia, and social anhedonia contributed to one factor, which we named the reflective self; magical ideation, perceptual aberration, and basic symptoms comprised the pre-reflective self factor. These two distinct factors were compatible with the previous idea of two aspects of the self and the characteristics of each level (41). Although basic symptoms were categorized as pre-reflective self due to the high loading on the factor (0.759), it also had meaningful loading on reflective self (0.460). This result showed that pre-reflective sense of self is an important foundation for reflective self, as indicated in a previous study (71). Four components of schizotypy were split into two factors: physical and social anhedonia in the reflective self factor; and perceptual aberration and magical ideation in the pre-reflective self factor. This finding may be explained by the characteristics of the questionnaires for physical and social anhedonia. Since the questionnaires required the subjects to describe how they feel in the face of hypothetical situations, intrinsic reflective selfhood might be reflected in the result of social anhedonia and physical anhedonia self-reports (72). The last factor was neurocognition; it included verbal memory, attention/working memory, psychomotor speed, executive function, and spatial memory. These domains constituted five important factors of neurocognition, with differences between the UHR group and the normal control in a previous study (29).

Factor scores differed among the three groups in this study. The social-cognitive bias score was higher in the UHR group than in the other two groups, yet not significantly so. This finding is not compatible with previous findings of bias in UHR (40, 73) and first-episode groups (73). However, it may be derived from the differences of hostility perception and blaming bias scoring; the AIHQ scoring in the previous study was related to the ambiguous situations, and not to the intentional and accidental ones. On the contrary, in this study, the AIHQ scoring was related to all three types of situations. The reflective self factor score was significantly different among the three groups. Since higher reflective self factor score implies lower self-esteem and resilience and higher anhedonia, the score of the HC group was the lowest of the three groups, as expected. This finding was compatible with previous reports of lower resilience and self-esteem in patient groups (38, 73). The UHR group showed higher reflective self score than the ROSPR group. This might suggest that individuals at UHR for psychosis experience more negative self-representation, and incomplete compensation occurs after progression toward overt psychosis. The neurocognition factor score was also significantly different among the three groups: the HC group had the highest score, followed by the UHR group and the ROSPR group. This result is in agreement with previous studies showing that patients with schizophrenia present lower neurocognitive function (3, 74, 75) and that individuals at UHR for psychosis already have neurocognitive impairments (29). The pre-reflective self factor score was significantly lower in the HC group than in the other two groups. Considering the high loadings of magical ideation, perceptual aberration, and basic symptoms of the pre-reflective self factor, the higher scores in the clinical groups were reasonable and compatible with previous studies showing higher basic symptoms and schizotypy scores in individuals at UHR for psychosis (39). Higher factor scores in the UHR and ROSPR groups are consistent with the concept that the basic symptoms of schizophrenia allow the identification of the earliest-experienced subjective symptoms (76).

Regarding the psychosocial function at baseline, multiple regression analysis of the four factors and QLS scores revealed the associated and predictive factors of psychosocial functioning. In individuals at UHR for psychosis, the total QLS score was negatively associated with SANS, social-cognitive bias, reflective self, and neurocognition factors. This finding was compatible with previous studies showing that lower social cognition and higher resilience are associated with better psychosocial functioning (37, 38) and that better neurocognitive performance is associated with higher total QLS scores (34). SANS, but not SAPS, was identified as an important factor affecting psychosocial function in the regression model. Negative symptoms are known to be associated with social functioning of individuals at UHR for psychosis and patients with schizophrenia (77). QLS subscores revealed correlations between each factor and social functioning, which was compatible with the results of the total QLS score. In the ROSPR group, the total QLS score was negatively associated with negative symptoms and the reflective self factor. This finding was compatible with a previous study showing that high resilience and self-esteem are correlated with better social functioning (20–22). Social cognitive bias, neurocognition, and pre-reflective factors were not predictive of psychosocial function in the ROSPR group. However, previous studies had suggested that social cognition (7–9) and neurocognition (2–4) were associated with social functioning. Neurocognition lost power for explaining QLS in the ROSPR group, probably due to its relationship with other highly affecting factors. Previous studies suggested that neurocognition in schizophrenia was related with the symptom domain, especially negative symptoms (78). Negative symptoms are a key factor affecting social functioning in schizophrenia (77, 79, 80). Considering previous studies and our results, negative symptoms could play a mediating role between social functioning and neurocognition in schizophrenia. SANS and the reflective self factor accounted for substantial variance of the QLS score; hence, other factors had probably lost their predictive power. Considering the results in the UHR and ROSPR groups, reflective self factor and SANS scores were associated with total, interpersonal relations, and intrapsychic foundation scores of QLS in the same pattern. These results may suggest that the reflective self factor is highly related with psychosocial function throughout the clinical course of schizophrenia. The finding that the pre-reflective factor was not associated with QLS probably indicates that the pre-reflective level of self was less associated with social functioning than the reflective level of self, yet there was not enough previous study about the association between social functioning and the pre-reflective aspect of self.

During follow-up, the neurocognition factor in the UHR group significantly predicted the conversion to overt psychosis in the Cox regression analysis. This finding was compatible with previous studies showing that low neurocognitive performance predicts higher conversion rate in the UHR group (29). In contrast to previous studies (31, 39), other factors failed to predict conversion. Among the social cognitive measures, we used AIHQ to measure social cognitive bias; however, previous studies suggested that the theory of mind, among social cognition, was related to conversion in the high-risk group (31). Including other domains in social cognition would increase the power of predicting the conversion of the UHR group. Therefore, further study is needed. Basic symptoms and schizotypy predicted conversion of UHR to overt psychosis in a previous study; however, in the present study, the pre-reflective and reflective self factors were not significant factors in the regression model. Considering the components of each factor, schizotypy was divided into two levels of self aspect, which can reduce the predicting power of each factor. One study suggested the predictive value of schizotypy; however, among the subscores, only physical anhedonia predicted conversion in the high-risk group (81). Another study failed to demonstrate basic symptoms and schizotypy as predicting factors for conversion when using combined variables (39). Further research is needed to determine the different effects of each component for predicting conversion. In the ROSPR group, meanwhile, the four factors failed to establish a statistically significant model to predict (re)admission. In a previous study on rehospitalization of patients with schizophrenia, 19 subjects were rehospitalized; this number was substantially higher than ours (*n* = 5) (5). Further studies with a larger sample could improve our knowledge about the predictive factors for (re)admission in patients with schizophrenia.

This study had several limitations. First, we could not establish any causal relationships of psychosocial function due to the cross-sectional design of the study and small sample size. A long-term study with a larger sample size could provide more information about this four-factor structure and its possible causal relationships with psychosocial function of individuals with ROSPR or at UHR for psychosis. Second, patients with ROSPR were mostly clinically stable and cooperated with the evaluation at baseline. Therefore, psychosocial functioning and other measures might have been underestimated at the moment of assessment. Subsequent regular follow-up could eliminate underestimation of the measures and increase the explanatory power of the results.

In conclusion, factor analysis revealed an intrinsic four-factor structure of social-cognitive bias, reflective self, neurocognition, and pre-reflective self. The four factors were associated with social functioning in the UHR and ROSPR groups at baseline and prodrome-to-psychosis conversion during follow-up in the UHR group. However, no factor was found to predict(re)admission in the ROSPR group. These findings indicate the clinical significance of the four-factor structure for patients with schizophrenia spectrum disorders, and provide a framework for understanding schizophrenia.

## Ethics Statement

This study was carried out in accordance with the Declaration of Helsinki. The Institutional Review boards at Severance Hospital reviewed and approved this study. All subjects, or the parents of subjects who were under 18 years old, gave written informed consent to participate in the study.

## Author Contributions

SA designed the study. SA, EL, JK, JP, and KK recruited subjects. HK undertook the statistical analysis and wrote the first draft of the manuscript. HK, HP, ES, MB, YS, SL, and KK interviewed patients and collected data. All authors contributed to revising the manuscript.

## Conflict of Interest Statement

The authors declare that the research was conducted in the absence of any commercial or financial relationships that could be construed as a potential conflict of interest.
